# Supplementary material for: Unveiling the Bioactive Potential of the Invasive Jellyfish Phyllorhiza punctata Through Integrative Transcriptomic and Proteomic Analyses
Source: Biomolecules. 2025 Aug 4;15(8):1121. doi: 10.3390/biom15081121 (PMC12383608; doi:10.3390/biom15081121)
Supplement: Supplementary file 1 [file biomolecules-15-01121-s001.zip › Suplementary Material/FileS3/PEPFOLD4/XP_057297449_1309/clusters.html]

 PEP-FOLD clusters


Download archive of all models  here   

|  |  |  |
| --- | --- | --- |
| # | file | sOPEP |
| # cluster 1 - Size: 2 |  |  |
|  | XP\_057297449\_1309-model1 | -34.9071 |
|  | XP\_057297449\_1309-model1.1 | -34.0802 |
| # cluster 2 - Size: 1 |  |  |
|  | XP\_057297449\_1309-model2 | -34.3625 |
| # cluster 3 - Size: 1 |  |  |
|  | XP\_057297449\_1309-model3 | -31.8283 |
| # cluster 4 - Size: 2 |  |  |
|  | XP\_057297449\_1309-model4 | -29.3668 |
|  | XP\_057297449\_1309-model4.1 | -21.6824 |
| # cluster 5 - Size: 8 |  |  |
|  | XP\_057297449\_1309-model5 | -28.975 |
|  | XP\_057297449\_1309-model5.1 | -26.9352 |
|  | XP\_057297449\_1309-model5.2 | -26.7178 |
|  | XP\_057297449\_1309-model5.3 | -26.6661 |
|  | XP\_057297449\_1309-model5.4 | -26.6208 |
|  | XP\_057297449\_1309-model5.5 | -26.2667 |
|  | XP\_057297449\_1309-model5.6 | -26.2214 |
|  | XP\_057297449\_1309-model5.7 | -25.2646 |
| # cluster 6 - Size: 1 |  |  |
|  | XP\_057297449\_1309-model6 | -27.6219 |
| # cluster 7 - Size: 4 |  |  |
|  | XP\_057297449\_1309-model7 | -27.0231 |
|  | XP\_057297449\_1309-model7.1 | -26.868 |
|  | XP\_057297449\_1309-model7.2 | -25.5554 |
|  | XP\_057297449\_1309-model7.3 | -24.6578 |
| # cluster 8 - Size: 7 |  |  |
|  | XP\_057297449\_1309-model8 | -26.7939 |
|  | XP\_057297449\_1309-model8.1 | -26.6719 |
|  | XP\_057297449\_1309-model8.2 | -26.3975 |
|  | XP\_057297449\_1309-model8.3 | -26.3542 |
|  | XP\_057297449\_1309-model8.4 | -25.9194 |
|  | XP\_057297449\_1309-model8.5 | -25.7273 |
|  | XP\_057297449\_1309-model8.6 | -25.4802 |
| # cluster 9 - Size: 3 |  |  |
|  | XP\_057297449\_1309-model9 | -26.5606 |
|  | XP\_057297449\_1309-model9.1 | -25.7333 |
|  | XP\_057297449\_1309-model9.2 | -25.602 |
| # cluster 10 - Size: 2 |  |  |
|  | XP\_057297449\_1309-model10 | -26.2589 |
|  | XP\_057297449\_1309-model10.1 | -25.0403 |
| # cluster 11 - Size: 1 |  |  |
|  | XP\_057297449\_1309-model11 | -25.8767 |
| # cluster 12 - Size: 1 |  |  |
|  | XP\_057297449\_1309-model12 | -25.7236 |
| # cluster 13 - Size: 3 |  |  |
|  | XP\_057297449\_1309-model13 | -25.5423 |
|  | XP\_057297449\_1309-model13.1 | -23.8449 |
|  | XP\_057297449\_1309-model13.2 | -22.3675 |
| # cluster 14 - Size: 2 |  |  |
|  | XP\_057297449\_1309-model14 | -25.5177 |
|  | XP\_057297449\_1309-model14.1 | -25.1141 |
| # cluster 15 - Size: 3 |  |  |
|  | XP\_057297449\_1309-model15 | -25.5082 |
|  | XP\_057297449\_1309-model15.1 | -21.7443 |
|  | XP\_057297449\_1309-model15.2 | -21.4841 |
| # cluster 16 - Size: 2 |  |  |
|  | XP\_057297449\_1309-model16 | -25.4559 |
|  | XP\_057297449\_1309-model16.1 | -25.2549 |
| # cluster 17 - Size: 7 |  |  |
|  | XP\_057297449\_1309-model17 | -25.4118 |
|  | XP\_057297449\_1309-model17.1 | -25.0271 |
|  | XP\_057297449\_1309-model17.2 | -24.8152 |
|  | XP\_057297449\_1309-model17.3 | -24.3674 |
|  | XP\_057297449\_1309-model17.4 | -24.2156 |
|  | XP\_057297449\_1309-model17.5 | -23.9578 |
|  | XP\_057297449\_1309-model17.6 | -23.7347 |
| # cluster 18 - Size: 5 |  |  |
|  | XP\_057297449\_1309-model18 | -25.3948 |
|  | XP\_057297449\_1309-model18.1 | -25.0438 |
|  | XP\_057297449\_1309-model18.2 | -24.7517 |
|  | XP\_057297449\_1309-model18.3 | -24.3819 |
|  | XP\_057297449\_1309-model18.4 | -24.3106 |
| # cluster 19 - Size: 1 |  |  |
|  | XP\_057297449\_1309-model19 | -25.3905 |
| # cluster 20 - Size: 3 |  |  |
|  | XP\_057297449\_1309-model20 | -25.3419 |
|  | XP\_057297449\_1309-model20.1 | -25.2788 |
|  | XP\_057297449\_1309-model20.2 | -24.1438 |
| # cluster 21 - Size: 1 |  |  |
|  | XP\_057297449\_1309-model21 | -25.3205 |
| # cluster 22 - Size: 3 |  |  |
|  | XP\_057297449\_1309-model22 | -25.2152 |
|  | XP\_057297449\_1309-model22.1 | -24.9555 |
|  | XP\_057297449\_1309-model22.2 | -24.597 |
| # cluster 23 - Size: 9 |  |  |
|  | XP\_057297449\_1309-model23 | -25.2017 |
|  | XP\_057297449\_1309-model23.1 | -24.7374 |
|  | XP\_057297449\_1309-model23.2 | -24.6933 |
|  | XP\_057297449\_1309-model23.3 | -24.5707 |
|  | XP\_057297449\_1309-model23.4 | -24.4808 |
|  | XP\_057297449\_1309-model23.5 | -24.4461 |
|  | XP\_057297449\_1309-model23.6 | -24.2079 |
|  | XP\_057297449\_1309-model23.7 | -24.1267 |
|  | XP\_057297449\_1309-model23.8 | -24.0413 |
| # cluster 24 - Size: 6 |  |  |
|  | XP\_057297449\_1309-model24 | -25.1572 |
|  | XP\_057297449\_1309-model24.1 | -24.7829 |
|  | XP\_057297449\_1309-model24.2 | -24.5765 |
|  | XP\_057297449\_1309-model24.3 | -24.5521 |
|  | XP\_057297449\_1309-model24.4 | -24.4785 |
|  | XP\_057297449\_1309-model24.5 | -23.7256 |
| # cluster 25 - Size: 2 |  |  |
|  | XP\_057297449\_1309-model25 | -24.976 |
|  | XP\_057297449\_1309-model25.1 | -24.5399 |
| # cluster 26 - Size: 3 |  |  |
|  | XP\_057297449\_1309-model26 | -24.8487 |
|  | XP\_057297449\_1309-model26.1 | -24.2989 |
|  | XP\_057297449\_1309-model26.2 | -23.1925 |
| # cluster 27 - Size: 1 |  |  |
|  | XP\_057297449\_1309-model27 | -24.8237 |
| # cluster 28 - Size: 1 |  |  |
|  | XP\_057297449\_1309-model28 | -24.7755 |
| # cluster 29 - Size: 1 |  |  |
|  | XP\_057297449\_1309-model29 | -24.4211 |
| # cluster 30 - Size: 1 |  |  |
|  | XP\_057297449\_1309-model30 | -24.3642 |
| # cluster 31 - Size: 1 |  |  |
|  | XP\_057297449\_1309-model31 | -24.2971 |
| # cluster 32 - Size: 1 |  |  |
|  | XP\_057297449\_1309-model32 | -23.9049 |
| # cluster 33 - Size: 1 |  |  |
|  | XP\_057297449\_1309-model33 | -23.7776 |
| # cluster 34 - Size: 1 |  |  |
|  | XP\_057297449\_1309-model34 | -23.7764 |
| # cluster 35 - Size: 1 |  |  |
|  | XP\_057297449\_1309-model35 | -23.6881 |
| # cluster 36 - Size: 1 |  |  |
|  | XP\_057297449\_1309-model36 | -23.504 |
| # cluster 37 - Size: 1 |  |  |
|  | XP\_057297449\_1309-model37 | -23.1559 |
| # cluster 38 - Size: 1 |  |  |
|  | XP\_057297449\_1309-model38 | -23.0724 |
| # cluster 39 - Size: 2 |  |  |
|  | XP\_057297449\_1309-model39 | -22.1013 |
|  | XP\_057297449\_1309-model39.1 | -21.8266 |
| # cluster 40 - Size: 2 |  |  |
|  | XP\_057297449\_1309-model40 | -22.0709 |
|  | XP\_057297449\_1309-model40.1 | -21.611 |
| # cluster 41 - Size: 1 |  |  |
|  | XP\_057297449\_1309-model41 | -22.027 |
|  |  |  |
